# Criterion validity of a self-reported web-based data collection tool to measure daily school travel in middle school children

**DOI:** 10.1186/s12889-026-27628-2

**Published:** 2026-05-11

**Authors:** Mathias Andersson, Elena Tseli, Anna-Karin Lindqvist, Annie Palstam

**Affiliations:** 1 Department of Medical Sciences, School of Health and Welfare, Dalarna University, Falun, Sweden; 2 Department of Neurobiology, Care Sciences and Society, Division of Physiotherapy, Karolinska Institutet, Huddinge, Sweden; 3 Department of Health, Education and Technology, Luleå University of Technology, Luleå, Sweden; 4 Sahlgrenska Academy, Institute of Neuroscience and Physiology, Department of Clinical Neuroscience, University of Gothenburg, Gothenburg, Sweden; 5 Department of Occupational Therapy and Physiotherapy, Sahlgrenska University Hospital, Gothenburg, Sweden

**Keywords:** Active school transport, Commuting, Transportation, Physical activity, Measurement methods

## Abstract

**Background:**

Various interventions have been developed with the intention to increase the amount of physical activity among children, including those promoting active school travel (AST). However, no gold standard currently exists for measuring different travel modes in AST. This study evaluates the criterion validity of a web-based data collection tool designed for children to self-report their school travel.

**Purpose:**

To assess the criterion validity of a web-based data collection tool for daily self-registration of commuting mode, time, and distance among middle school children in Sweden.

**Methods:**

Thirty children (10–12 years old) from six schools in Falun, Sweden, were recruited using snowball sampling. The children self-reported their school travel data for one day, including travel mode, commuting time, and distance. Their reports were compared to a criterion standard based on direct observations. Spearman correlation and the Wilcoxon signed-rank test were used to analyze the accuracy of the self-reported data.

**Result:**

The web-based data collection tool demonstrated 100% agreement between self-reported and observed travel modes, with high correlations for commuting time and distance (r_s_=0.953–0.989, *p* < 0.001). The Wilcoxon signed rank test showed no significant differences between self-reported data and criterion standard (*p* = 0.243–0.903).

**Conclusion:**

The strong agreement between self-reported and observed travel data indicates high criterion validity, suggesting that the web-based data collection tool is a reliable method for middle school children to self-report their daily school travel.

## Background

The World Health Organization (WHO) recommends that children and adolescents engage in an average of 60 min of physical activity per day [1]. Since a majority of children worldwide do not meet these public health recommendations [1] various interventions have been developed to increase the amount of physical activity among children, amongst them interventions involving active school travel (AST) [2]. AST is defined as physically active commute (non-motorized travel, i.e., walking and cycling) when traveling to/from school, and different quantitative outcome measures that focus on the direct impact on physical activity have been used to evaluate the effect of AST interventions. Monitor-based evaluation measures like Global Positioning System-trackers (GPS trackers) [3, 4], accelerometers [4], or heart rate monitors [5] often use calculations of data for quantitative evaluation of AST interventions, and outcomes relevant to AST-interventions can also be evaluated using report-based measures like questionnaires [6], where weekly retrospective reportings are common [7, 8].

Presently, there is no gold standard to measure different modes of travel during AST, since the measuring methods display both advantages and disadvantages [8–12]. For example, monitor-based GPS trackers collect data on position, distance and speed, whereas modes of travel are assumed based on calculations of the relationships between these three parameters [4]. The accelerometer is another monitor-based objective measurement method that is considered valid when estimating children’s physical activity in relation to bodily movement [4, 9], with the exception of certain forms of activities, amongst them cycling [10, 11]. The exception of cycling is especially unfortunate in the Nordic countries where bicycle-borne school travel is very common [13, 14]. Hence, some authors use a combination of different monitor-based measurement methods to gather more comprehensive information on physical activity related to activity mode, time, and distance [15] and a combination of GPS trackers and accelerometers can therefore be used for evaluation of physical activity related to cycling to school [3, 16]. These monitor-based measurement methods require manual handling (distributing/collecting equipment), the downloading and interpretation of data, and the maintenance of the technical devices (including recharging the batteries). In comparison, report-based measurement methods, such as questionnaires, are often easier to scale and, hence, are associated with greater feasibility than monitor-based methods, but come with the cost of lower validity and increased risk of recall bias [7] when estimating AST and/or physical activity [6].

As present measurements display both advantages and disadvantages [4, 8], research on valid and feasible data collection methods, or a combination of device-based and self-reported measurement methods, to assess commuting to/from school has been asked for [4]. For this purpose, a web-based data collection tool for children to self-report daily school travels was developed, inspired by a weekly questionnaire on AST developed in Spain [12]. Throughout the development process, children actively contributed by providing input, helping to enhance user-friendliness to ensure that the instrument was as tailored as possible to the needs and preferences of the intended users. The children provided input on instructional texts, figures, and colouring.

The web-based tool for data collection enables the involvement of the children in their reporting of data (first-hand source), and the reporting in temporal proximity to the travel partially reduces the risk of recall or information bias. The online function enables a scalable digital distribution that can increase the precision of device-based measurement methods or be used as a standalone data collection method when quantity is considered “a quality”.

This study aimed to assess the criterion validity of the web-based data collection tool among middle school children in Sweden, and, feasibility and implementation is further to be tested.

## Methods

### Study design

This cross-sectional observational study of criterion validity assessed the agreement of children’s self-reported data in a web-based data collection tool compared with data from direct observations made by an accompanying data collector as criterion standard.

### Item of evaluation

**Fig. 1:**
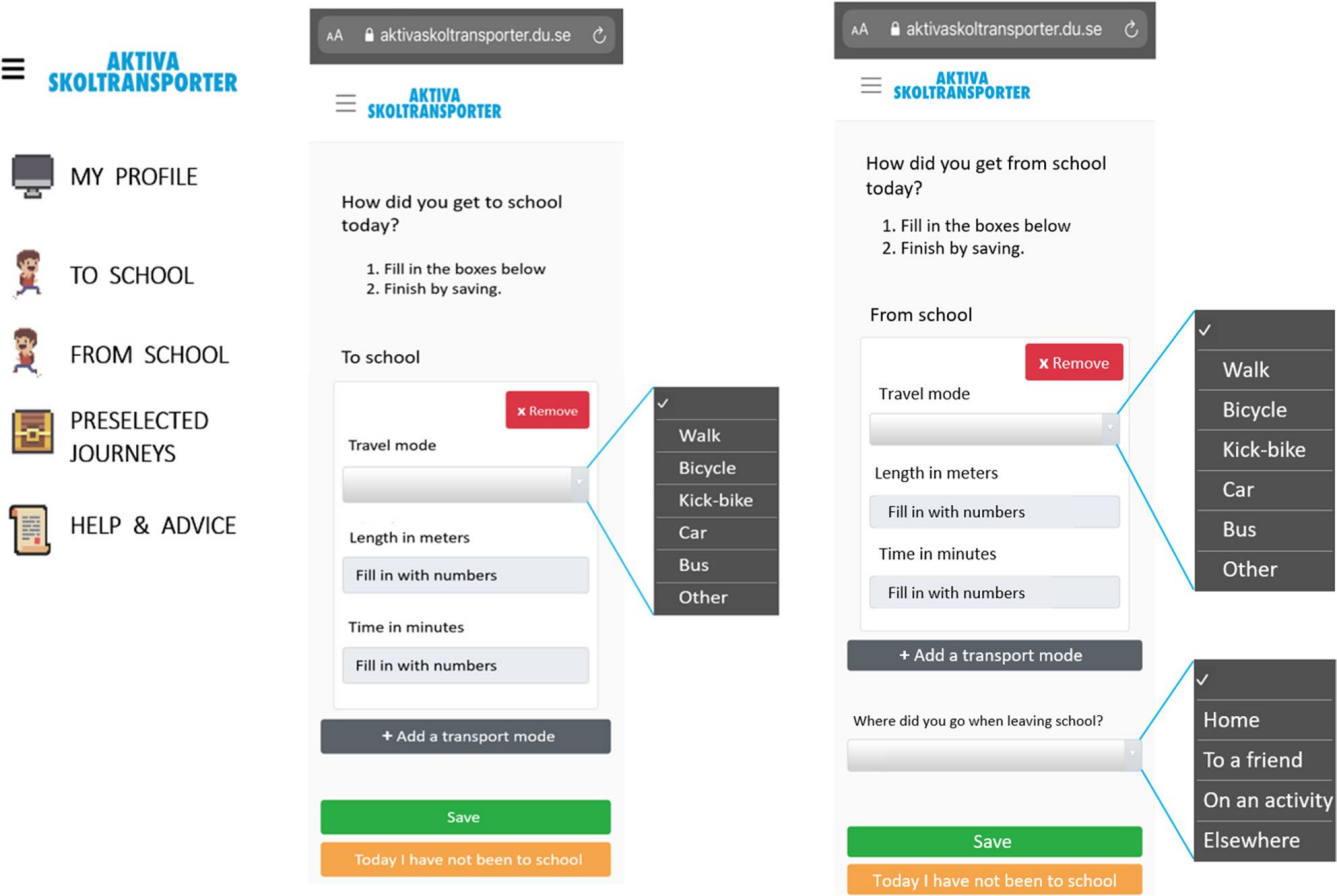
The view of the menu in the web-based data collection tool (left figure) and the view for reporting travel mode, commuting time and distance to and from school (middle and right figure)

The web-based data collection tool included three questions on daily school travels. First, a multiple-choice question on transport mode with fixed alternatives: walking, bicycle, kick-scooter, bus, car or other (other = e.g. skateboard, motor-assisted vehicle, tram, boat, etc.). Second, approximated distance travelled in meters, and third, approximated time travelled in minutes. The questions were posed separately regarding the trip to school and the trip from school for each day. Further, the tool allowed for use of split trips, for example when walking to the bus stop and then taking the bus to school from there, with the possibility of adding trips. When reporting commutes from school there was an additional multiple-choice question on the destination (Fig. 1).

### Recruitment and study sample

Thirty children from grades 4–6 (aged 10–12 years old) were recruited to report data from one day’s travel (to and from school) in the web-based data collection tool. The children were recruited from six different schools in Falun municipality, using snowball sampling performed by two data collectors. The majority of children were recruited when showing interest in the research activity in the schoolyard area or from participating children who suggested friends who might be interested in participating in the research. The children got verbal and written information about the research to provide to their caretaker for consent, and after approval from caretaker, the children shared their caretakers’ telephone numbers for the researcher to contact in order to discuss further arrangements. When presenting the research for the caretakers, some adults gave recommendations on names and mobile numbers to other potential families to contact. The number of children included was based on the recommended adequate sample size calculation of criterion validity [17].

### Procedure and data collection

After recruitment, one of the data collectors contacted the children via their caregivers’ telephone one to three days before the day of data collection to register name, gender, age, address, and school, and gave instructions on login and reporting of school travel in the web-based data collection tool. The data collector clarified that both travel mode, commuting time and distance were key in registering data in the web-based data collection tool, and that data was standardized to be measured in relation to the child’s place of departure and arrival, both on the commutes to and from school. The child was, if needed, instructed on how to measure time and distance. Information on how to report travels in the web-based data collection tool and how to measure time and distance were additionally presented in videos on a research project website together with text information on the execution of the study (step-by-step), the handling of data, and other specifics relevant to the research project.

After giving instructions, the data collector continued by registering the child’s mobile phone number in the web-based data collection tool and set the time for automatic delivery of a Short Message Service (SMS) with a web link to the tool, on the afternoon/evening on the pre-selected observation day. Further, the child, sometimes in consultation with their caretakers, stated the intended travel mode on the forthcoming day of data collection and agreed on a departure time. On the observation day, the data collector met the child at their residence and accompanied the child to school, regardless of traveling mode. As criterion standard, the data collector carefully noted the travel mode used by the child, the departure time from the child’s residence and the arrival time at the school for calculating the commuting time, and the travel routes were carefully registered for the succeeding measurements of distance, which were conducted using Google Maps. When arriving at the schoolyard area, the data collector and child agreed on a specific time to meet up after school, for repeating the same procedure on their way from school.

Later, in the afternoon or evening on the day of data collection, the children received the SMS at the pre-selected time with a link to the web-based data collection tool for login and reporting travels to and from school.

### Data analysis

Descriptive statistics were used to describe the characteristics of the children (age and gender). Reported travel mode, registered as walk/bicycle/kick-scooter/car/bus/other, was presented in percentage of agreement related to the criterion standard. Time and distance between the children´s residence and their school were presented with median, min-max, and interquartile range (IQR).

Spearman correlation was used to analyze the association between the children’s self-reported data and the criterion standard on time and distance. Correlation coefficients (r_s_) were considered as follows: negligible r_s_=0.00-0.30, low r_s_=0.30-0.50, moderate r_s_=0.50-0.70, high r_s_=0.70-0.90, and very high r_s_=0.90 − 1.0 [18, 19]. The Shapiro-Wilk test was used to test if the data was normally distributed, where a cut-off for non-normally distributed data was set at < 0.95. Because the distributions of measurement differences were not normal and the sample size was limited (*n* = 30), the non-parametric Wilcoxon signed-rank test was used to analyze the distribution of the differences between the self-reported data on time and distance and the criterion standard. A p-value less than 0.05 was considered statistically significant.

The median of values of children´s reports and criterion standards on time to school, time from school, distance to school, and distance from school were plotted using the Bland-Altman analysis [20, 21] to visually detect systematic differences. Again, due to the non-normal distribution of measurement differences and limited sample size, we used non-parametric calculations to estimate limits of agreement (LoA). The LoA were defined as the 2.5th and 97.5th percentiles of the differences and we used linear interpolation between adjacent order statistics to obtain percentile estimates.

Spearman correlation was used to analyze associations between the differences in children´s self-reports and criterion standard depending on time and distance. If any child was unable to participate, missing data was prevented by continuing the recruitment of children until 30 participants were reached. Quantitative data were analyzed using the Statistical Package Software for the Social Sciences (SPSS version 28.0.1.0, Chicago, IL, USA).

## Results

In total, 30 children participated in the study, of whom 19 were girls and 11 were boys and 17 were in fourth grade, 3 were in fifth grade and 10 were in sixth grade. Out of a maximum of 60 reported school trips, one was missing due to one child reporting only the trip to school, leaving a total of 59 reported school trips. The most frequently used transport mode was walking, which was used in 29 (49%) of children’s reports. Bicycling was used in 18 (31%), cars in 10 (17%) and buses in 2 (3%) of children’s reports.

No child used public transport for travel. All children reported their home as the destination of their travel from school. The median distance between home and school was 1170 m, ranging from 318 m up to 17,200 m, as registered by the accompanying data collector.

There was an absolute agreement between the children’s reported travel mode in the web-based data collection tool and criterion standard, where the reported transport modes were 100% matched with the direct observations registered as walk/bicycle/kick-scooter/car/bus/other. No child expressed a need for instructions on how to measure time, but all children were interested in getting instructions on how to measure distance using GoogleMaps™.

Correlations between the children´s reports and criterion standard on time and distance were high/very high, r_s_=0.953–0.989 (*p* < 0.001) (Table 1). Data was non-normally distributed with a W < 0.95 (*p* < 0.001) and the Wilcoxon signed rank test showed no differences between reports and criterion standard (*p* = 0.243–0.903) (Table 1).

**Table 1:** The analysis results from children´s reported data in the web-based data collection tool and data from criterion standard, and the relationship between the data

	Median minutes or meters (min-max) IQR	Spearman rank correlation (*r*_s_)	Reports vs. Criterion Lower/Identical/Higher	Wilcoxon signed rank test (z)
Time to school(*n*= 30)		0.953 (*p* < 0.001)	3 / 17 / 10	-1.66
Reported data, minutes	8.5(2–30)10			(*p* = 0.243)
Criterion standard, minutes	10(2–23)10			
Time from school (*n* = 29)		0.974 (*p* < 0.001)	7 / 12 / 10	-0.12
Reported data, minutes	8(2–30)11			(*p* = 0.903)
Criterion standard, minutes	9(2–26)10			
Distance to school (*n* = 30)		0.989 (*p* < 0.001)	17 / 0 / 13	-0.47
Reported data, meters	1210(307-17000)1154			(*p* = 0.636)
Criterion standard, meters	1170(318-17200)1302			
Distance from school (*n* = 29)		0.985 (*p* < 0.001)	12 / 1 / 16	-0.33
Reported data, meters	1200(150-17000)968			(*p* = 0.741)
Criterion standard, meters	1085(220-17200)939			

*IQR*  Interquatrile range

**Fig. 2:**
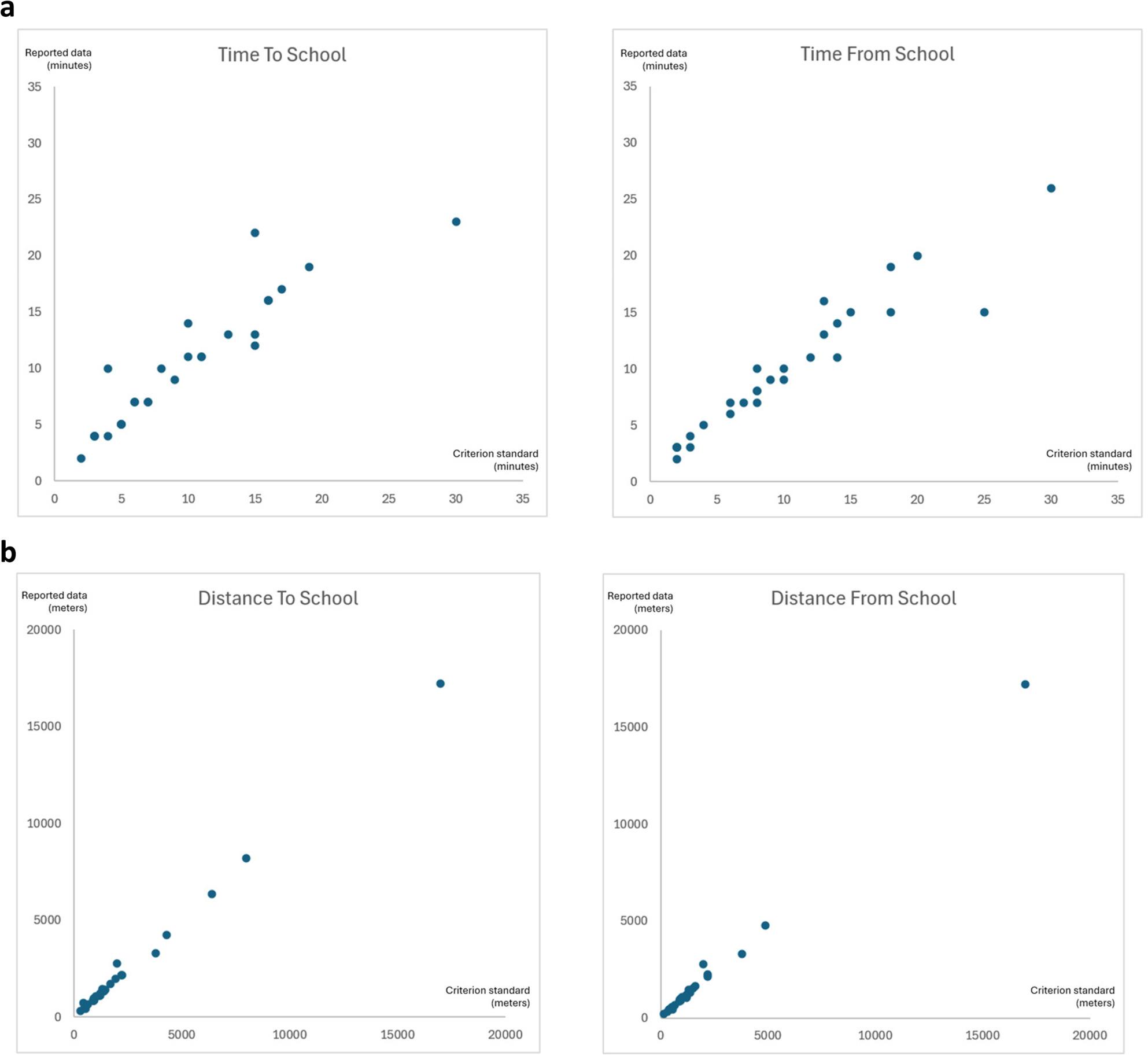
**a** Scatter plots on children´s reports on time in minutes (Y-axis) in relation to criterion standard (X-axis). **b** Scatter plots on children´s reports on distance in meters (Y-axis) in relation to criterion standard (X-axis)

Scatter plots visualizing the relationship between children´s reports and criterion standard on time to school and time from school are presented in Fig. 2a, and scatter plots on distance to school and distance from school are presented in Fig. 2b.

**Fig. 3:**
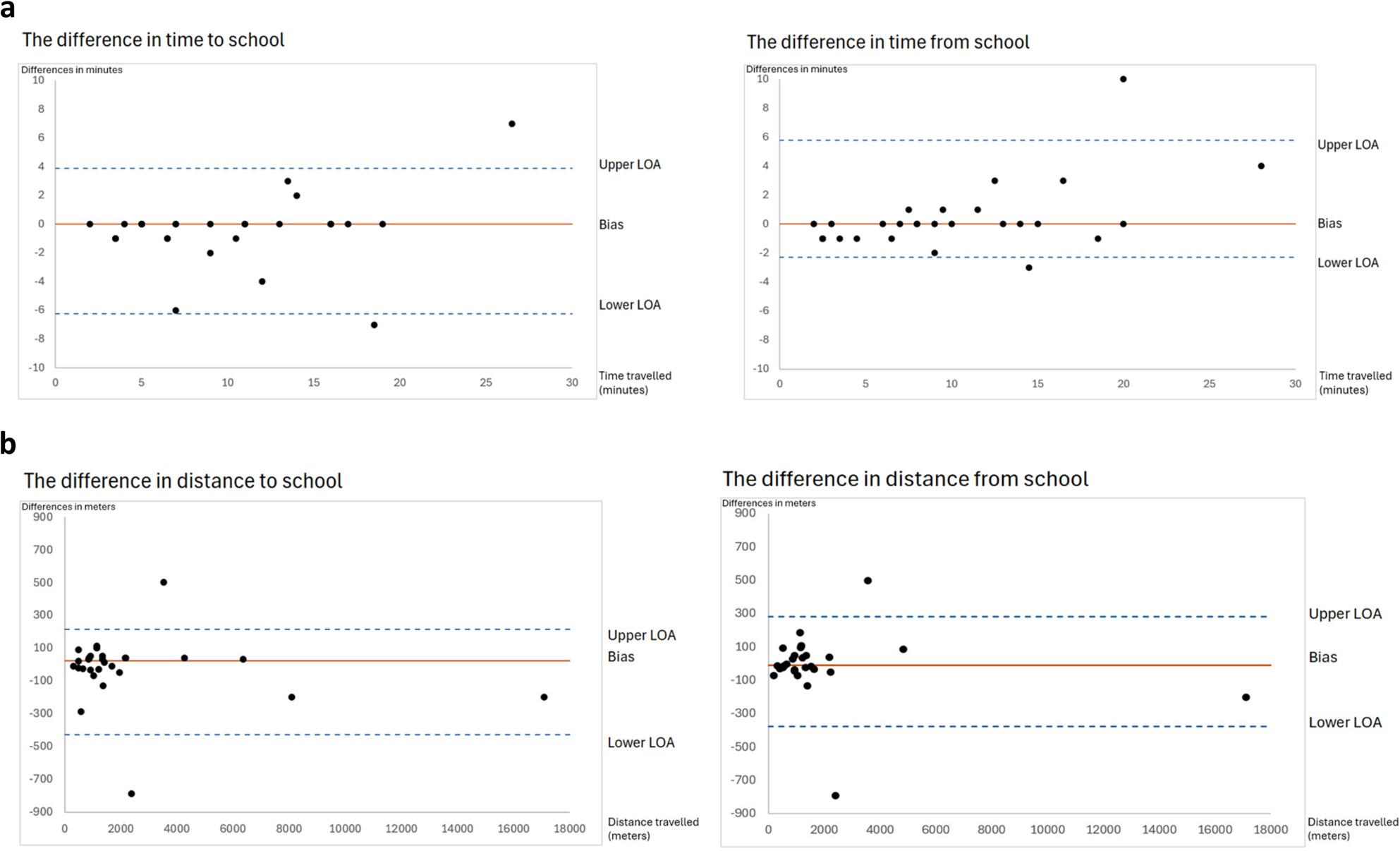
**a** Bland-Altman plots visualizing the agreement between median differences in measurements on time in minutes (Y-axis) in relation to median time travelled to- and from school in minutes (X-axis), presented with 2.5% and 97.5% nonparametric limits of agreement (LOA). Upper LOA in time to school = 4.1 minutes (min), Lower LOA = -6.275 min, and Bias = 0 min. Upper LOA in time from school = 5.8 min, Lower LOA = -2.3 min, and Bias = 0 min. **b** Bland-Altman plots visualizing the agreement between median differences in measurements of distance in meters (Y-axis) in relation to median distance travelled to- and from school (X-axis), presented with 2.5% and 97.5% nonparametric limits of agreement (LOA). Upper LOA in distance to school = 217.25 meters (m), Lower LOA = -427.5 m, and Bias =25 m. Upper LOA in distance from school = 262.3 m, Lower LOA = -377 m, and Bias =-10 m

Agreement between median differences in measurements to and from school was plotted using a Bland-Altman analysis with 2.5 and 97.5% nonparametric limits of agreement on time (Fig. 3a) and on distance (Fig. 3b). The correlations between commuting time and median differences in measurements on commuting time to, and from, school were negligible r_s_=0.185 (*p* = 0.328) and low to moderate r_s_=0.373 (*p* = 0.046) respectively. Correlations between commuting distance and median differences in measurements on commuting distance to and from school were negligible, r_s_=-0.098 (*p* = 0.608) and r_s_=-0.029 (*p* = 0.881), respectively.

## Discussion

This study aimed to evaluate the criterion validity of a web-based data collection tool for daily report of school travels in middle school children. The results showed an absolute agreement between reported travel mode and the criterion standard. Also, reports on time to- and from school showed very high correlations with the criterion standard. Reported time was identical with the criterion standard in half of the reported travels, and most of the remaining data on time differed by approximately one minute. Further, reported distance to- and from school showed very high correlations with the criterion standard, where the majority of the data had a difference up to 50 m (travels to school ~ 63.3%, and travels from school ~ 58.6%), overall indicating high criterion validity. However, there seemed to be a slight risk of systematic bias depending on the time travelled, as a longer travel time showed a low to moderate correlation with a larger difference between reports and the criterion standard, on the way home from school.

These findings of high criterion validity in self-reported transport mode in children are in line with the findings of a Spanish study evaluating the convergent validity of a weekly questionnaire on school transport [12]. However, the strong correlations found in the present study between reported time to- and from school and the criterion standard contrasts with the findings in the Spanish study, where the reported transport time in children aged 7–11 years was found to differ significantly from objectively measured transport time [12]. However, in adolescents aged 12–19, criterion validity was found to be satisfactory [12], in line with the results of the present study. The discrepancies in accuracy between children in different age groups need to be considered, since the result in this study only relate to 10–12 year old children. Also, findings relate to measurements performed during one school day, without aspects incident to consecutive data collection measurements.

Still, the web-based data collection tool can be used as presented in this study if all three reportings are required (travel mode, time and distance), or be modified for younger children to more easily make reports only related to data on travel mode. If modified, the dichotomization of commutes into “active travel” or “non-active travel” might more easily be generalized to a wider population of children due to less comprehensive instructions and reporting. If combined, a software modification with an additional data input may be supplemented enabling children to report starting time on their commutes for assisting the differentiation of commute-related and non-commute-related activities in the monitor-based data output analysis [4, 8–12].

Although there was no significant reporting bias for time nor distance to school, our results showed overestimations on longer transport times when traveling from school. This may relate to the findings from a qualitative study in which children described a tendency to choose the most direct route and to be aware of time costs when their travel route deviated from the planned path on their way to school, while feeling a greater sense of freedom on their way home [22]. The children stopped at the bicycle parking area to talk and play and did not seem to be in a hurry when leaving the schoolyard. Also, all children then traveled straight to their homes after school in this study, not engaging in spontaneous activities or alternative travel paths, although some children mentioned that they often could take detours or go straight to their friends’ house on their way home from school. It could be anticipated that such activities were actively avoided by the children on the day of the measurements, as they were aware of being a part of a study meanwhile being observed by a data collector, altogether potentially influencing their travel behavior. The same aspect can be related to the finding that one child missed reporting commutes from school, and, hence, reporting on commutes to school might be considered the primary outcome when evaluating the effects of AST interventions. This is an issue worth consideration when designing future studies evaluating validity of AST measures.

The login procedure in the web-based data collection tool connects the data to the respective child and the individualized data can therefore be used to connect reported data to other reporting e.g., from caretakers or school administration. This interconnection can, hypothetically, be used to address wider research questions or can be seen as an unwanted intrusion that advocates alternative anonymized reporting procedures.

A practical relevance in using the web-based data collection tool is that the data also can be used to estimate energy expenditure in addition to the performed physical activity. This estimation could provide opportunities for quantifying and evaluating health aspects of AST [4]. Yet, if there is a high demand for accuracy in data on specific activity levels, report-based measurement methods can beneficially be combined with objective monitor-based measurement devices [4, 9, 23].

### Strengths and limitations of the study

In total, 30 children were included in the study, which permitted separate analyses of the data to- and from school, still reaching adequate sample size for analyses of criterion validity [17]. This is a strength of the study since the separation of trips to- and from school enabled a more detailed analysis and generated more specific results of validity, considering possible differences in transport behavior and reporting accuracy depending on these transports, respectively. Using direct observations is another strength of the study, since this method circumvents other measurement reported assumptions of the exact starting and stopping time in relation to departure and arrival. However, since the children knew they were being observed, they probably were more fastidious when measuring their trips and reporting data. If trips are to be reported over multiple days in a school week, there is a risk of measurement error related to nonchalant reporting and/or with duplications of data from previous recordings.

Although the accuracy when using this method in future studies might be questionable without the added possible Hawthorne effect related to the accompanying researcher, the result still show that children (10–12 years old) have the ability to measure and report data from their trips without assistance from researchers or teachers. A caveat worth noting is that discrepancies between reported and observed values increased with longer travel times on the way home from school, which may limit the precision of the tool for longer or more variable commutes.

One limitation of the study concerns the instructions given to the children on estimations of time and distance. Time and distance to and from school were standardized to be measured in relation to the child’s residence and the schoolyard area, and the children were provided with general instructions on how to manage the measurement function in GoogleMaps™. However, no explicit instructions were given on the accuracy of the placement of measurement markings in the actual map. The same approach was applied when instructing the children how to estimate time in minutes, where the children’s choice on how to measure commuting time may have resulted in different measurement accuracy. However, given that most of the reported travel times and distances had very high accuracy it could be assumed that no specific instructions are needed, which implies opportunities for the feasibility of larger scale studies using the web-based data collection tool, without compromising accuracy in reports.

Also, the children were given the option to choose when they preferred to receive the link to the web-based data collection tool. This non-standardized flexibility on response time can be considered a potential recall bias for those who set the time to a late hour in the evening, but the option to individualize the direct reports from the firsthand source, the child, can also be recorded in more temporal proximity to the trips and be considered a reduction of information bias. Further, the recruitment procedure, together with participation being conducted out of school hours, may have attracted children interested in research. This may have entailed a recruitment bias linked to the reporting [24, 25] as children signing up for the study can be considered more prone to participate and engage in precise reporting.

Report-based measurement methods are associated with high feasibility, at the cost of lower validity, when estimating levels of physical activity in children [6], and the distributions to greater populations are, hence, considered possible as digital video conferences can replace individual telephone instructions. This can enable several children to simultaneously access the web-based data collection tool in an i.e. classroom setting and thereby be considered a strength regarding the usability and generalizability of the web-based data collection tool. However, awareness that the authors were engaged in the previous development of the web-based data collection tool can be considered a bias that might have impacted the results primarily on how 10–12-year-old children were able to report data after only taking part in a short instruction via telephone.

## Conclusion

The absolute agreement between the children’s reported travel mode and criterion standard as well as the very high correlations between reports on time and distance and criterion standard all indicate a high criterion validity of the web-based data collection tool. In addition, the small biases observed between reported and observed outcomes further support the tool’s criterion validity. This implies that the web-based data collection tool can be considered a valid method to self-report daily school travel by children in middle school.

## Data Availability

The data analyzed in the current study are not publicly available due to ethical restrictions. According to the Swedish regulations ( https://etikprovningsmyndigheten.se/ , accessed 20 November 2024), permission to use data is only for the purpose for which it has been approved by the Swedish Ethical Review Authority. Data requests can be made to dataskydd@du.se. Point of contact (AP) apl@du.se.
